# Twenty years of research on borderline personality disorder: a scientometric analysis of hotspots, bursts, and research trends

**DOI:** 10.3389/fpsyt.2024.1361535

**Published:** 2024-03-01

**Authors:** Yuanli Liu, Chaomei Chen, Ying Zhou, Na Zhang, Shen Liu

**Affiliations:** ^1^ Department of Psychology, School of Humanities and Social Sciences, Anhui Agricultural University, Hefei, China; ^2^ College of Computing & Informatics, Drexel University, Philadelphia, PA, United States; ^3^ Department of Psychology, School of Education, China University of Geosciences, Wuhan, China; ^4^ Department of Information Management, Anhui Vocational College of Police Officers, Hefei, China

**Keywords:** neuro-behavioral model, borderline personality disorder, BPD, bibliometric, Scimago Graphica

## Abstract

Borderline personality disorder (BPD), a complex and severe psychiatric disorder, has become a topic of considerable interest to current researchers due to its high incidence and severity of consequences. There is a lack of a bibliometric analysis to visualize the history and developmental trends of researches in BPD. We retrieved 7919 relevant publications on the Web of Science platform and analyzed them using software CiteSpace (6.2.R4). The results showed that there has been an overall upward trend in research interest in BPD over the past two decades. Current research trends in BPD include neuroimaging, biological mechanisms, and cognitive, behavioral, and pathological studies. Recent trends have been identified as “prevention and early intervention”, “non-pharmacological treatment” and “pathogenesis”. The results are like a reference program that will help determine future research directions and priorities.

## 1 Introduction

Borderline personality disorder (BPD) is a complex and severe psychiatric disorder characterized by mood dysregulation, interpersonal instability, self-image disturbance, and markedly impulsive behavior (e.g., aggression, self-injury, suicide) (1). In addition, people with BPD may have chronic, frequent, random feelings of emptiness, fear, and so on. These symptoms often lead them to use unhealthy coping mechanisms in response to negative emotions, such as alcohol abuse (2). BPD has a long course, which makes treatment difficult and may have a negative impact on patients’ quality of life (3). Due to its clinical challenge, BPD is by far the most studied category of personality disorder (4). This disorder is present in 1−3% of the general population as well as in 10% of outpatients, 15−20% of inpatients, and 30−60% of patients with a diagnosed personality disorder, and has a suicide rate of up to 10% (5, 6). Families of individuals with serious mental illness often experience distress, and those with relatives diagnosed with BPD tend to carry a heavier burden compared to other mental illnesses (7, 8). As early as the 20th century, scholars began describing BPD and summarizing its symptoms. However, there was some debate regarding the precise definition of BPD.

In the past few decades, the research community has made remarkable progress in the study of BPD, equipping us with a wider range of perspectives and tools for understanding this intricate condition. However, numerous challenges still remain to be tackled by researchers. Diagnosing BPD is inherently challenging and often more difficult than anticipated. The symptoms of BPD are complex, diverse, and often overlap with those of other mental health conditions. For example, individuals with BPD may experience extreme mood swings similar to those observed in individuals with bipolar disorder (9); At the same time, they may also be entrenched in long-term depression, making it easy for doctors to initially misdiagnose them with depression (10). Because these symptoms overlap and interfere with each other, doctors often face the risk of misdiagnosing or overlooking the condition during initial diagnosis. Therefore, researchers are working to develop more accurate and comprehensive diagnostic tools and methods.

According to the “Neuro-behavioral Model” proposed by Lieb (1), the process of BPD formation is very complex and is determined by the interaction of several factors. The interaction between different factors can be complex and dynamic. Genetic factors and adverse childhood experiences may contribute to emotional disorders and impulsivity, leading to dysfunctional behaviors and inner conflicts. These, in turn, can reinforce emotional dysregulation and impulsivity, exacerbating the preexisting conditions. Genetic factors are an important factor in the development of BPD (11). Psychosocial factors, including adverse childhood experiences, have also been strongly associated with the development of BPD (12). Emotional instability and impulsive behavior are even more common in patients with BPD (13). The current study is based on the “Neuro-behavioral Model” and conducts a literature review of previous scientific research on BPD through bibliometric analysis to reorganize the influencing factors. Through large-sample data analysis, the association between BPD and other diseases is explored, which contributes to further refining this theory’s explanation of the common neurobiological mechanisms among various mental illnesses.

It is worth noting that with the development of BPD, some scholars have conducted bibliometrics studies on BPD to provide insights into this academic field. To date, the current study has identified two published bibliometric studies on the field: One is Ilaria M. A. Benzi and her colleagues’ 2020 metrological analysis of the literature in the field of BPD pathology for the period 1985−2020 (14). The other is a bibliometric analysis by Taylor Reis and his colleagues of the growth and development of research on personality disorders between 1980 and 2019 (15). Ilaria M. A. Benzi and her colleagues integrated and sorted out the research results of borderline personality pathology, and revealed the research results and development stages in this field through the method of network and cluster analysis. The results of the study clearly demonstrate that the United States and European countries are the main contributors, that institutional citations are more consistent, and that BPD research is well developed in psychiatry and psychology. At the same time, the development of research in borderline personality pathology is demonstrated from the initial development of the construct, through studies of treatment effects, to the results of longitudinal studies. Taylor Reis and his colleagues used a time series autoregressive moving average model to analyze publishing trends for different personality disorders to reveal their historical development patterns, and projected the number of publications for the period 2024 to 2029. The study finds a trend towards diversity in the research and development of personality disorders, with differences in publication rates for different types of personality disorders, and summarizes the reasons that influence these differences. This may ultimately determine which personality disorders will remain in future psychiatric classifications. These studies have provided valuable insights into the evolution of BPD, focusing primarily on its pathology or a broader personality disorder perspective. While basic bibliometric analyses of these studies have been conducted, there is a need for more in-depth investigations of specific trends in the evolution of BPD and a clearer delineation of emerging research foci. Therefore, in order to enhance the current study, this study extends the analysis to 2022 and utilizes a comprehensive structural variation analysis of the literature using scientometric methods. Building on previous bibliometric studies, we expect to provide new insights and additions to research in this area. At the same time, the research trends and hot topics in the field of BPD are further explored. In addition, several cocitation-based analyses are also carried out in order to better understand citation performance.

## 2 Methods

### 2.1 Objectives

One of our goals was to understand the current status and progress of researches on BPD, and to summarize the latest developments and research findings in BPD, such as new treatment methods and disease mechanisms. Through the intuitive presentation of knowledge graphs and other images or data, we aimed to provide clinical practice and research guidance for clinicians, researchers, and policymakers.

Our second goal was to help identify future research directions and priorities, and provide more scientific and systematic research guidance for researchers. For example, by identifying hotspots and associations in certain research areas, we can determine the fields and issues that require further investigations, thus providing clearer directions and focus for researches. Additionally, through bibliometric analysis, we can provide researchers with more targeted and practical research strategies and methods, improving research efficiency and the quality of research outcomes.

### 2.2 Search strategy and data collection

The selection of appropriate methods and tools in the process of analyzing research information is crucial. Web of Science (WOS) is a popular database for bibliometric analysis that includes numerous respectable and high-impact academic journals. In addition, data information, such as references and citations, is more extensive than other academic databases (16). Data collection took place on the date of May 10, 2023. The search strategy included the following: topic=“Neuro-behavioral Model” or “borderline characteristics” or “borderline etiology” or “borderline personality disorder”, database selected=WOS Core Collection, time span=2003−2022, index=Science Citation Index Expanded (SCI-EXPENDED) and Social Sciences Citation Index (SSCI). The “Neuro-behavioral Model” serves as a theoretical framework that is useful for explaining the development and pathophysiology of BPD; “borderline characteristics” can describe the related symptoms and features of BPD; “borderline etiology” helps to understand the factors that contribute to the development of BPD; “borderline personality disorder” is the most commonly used terms in relevant research. Using these as keywords in title searches can help researchers find researches related to BPD more accurately, facilitating deeper understanding of the characteristics, pathophysiology, etiology, and other aspects of BPD. In the current study, we focused only on two types of literature: articles and review articles, and limited the language to English. After removing all literature unrelated to BPD, a total of 7919 records met the criteria. They were exported in record and reference formats, and saved in plain text file format.

### 2.3 Data analysis and tools

Bibliometrics was first proposed by Alan Pritchard in 1969, as a method that combines data visualization to analyze publications statistically and quantitatively in specific fields and journals (17). Bibliometric analysis is a good way to analyze the trend of knowledge structure and research activities in scientific fields over time, and has been widely used in various fields since it was first used (18). Scientometrics is the application of bibliometrics in scientific fields, and it focuses on the quantitative characteristics and features of science and scientific researches (19). Compared to traditional literature review studies, visualized knowledge graphs can accurately identify key articles from many publications, comprehensively and systematically combing existing research in a field (20).

Currently, two important academic indicators are included in research. The impact factor (IF) is used as an indicator of a publication’s impact to assess the quality and importance of the publication (21). However, some researchers believe that IF has defects such as inaccuracy and misuse (22). Although many researchers have proposed to replace the impact factor with other indicators, IF is still one of the most effective ways to measure the impact of a journal (23). The IF published in the 2021 Journal Citation Reports were used. Another indicator is the H-index, which is an important measure of a scholar’s academic achievements. Some researchers consider it as a correction or supplement to the traditional IF (24).

All data were imported into CiteSpace (6.2.R4) and Scimago Graphica (1.0.30) for analysis. CiteSpace was used to obtain collaboration networks and impact networks. Scimago Graphica was used to construct a network graph of country collaboration. CiteSpace is a Java-based software developed in the context of scientometrics and data visualization (25). It combines scientific knowledge mapping with bibliometric analysis to determine the progress and current research frontiers in a particular field, as well as predict the development trends in that field (26). Scimago Graphica is a no-code tool. It can not only perform visualization analysis on communication data but also explore exploratory data (27). Currently, it is used for visual analysis of national cooperation relationships, displaying the geographic distribution of countries and publication trends.

## 3 Results

### 3.1 Analysis of publication outputs, and growth trend prediction

**Figure 1:**
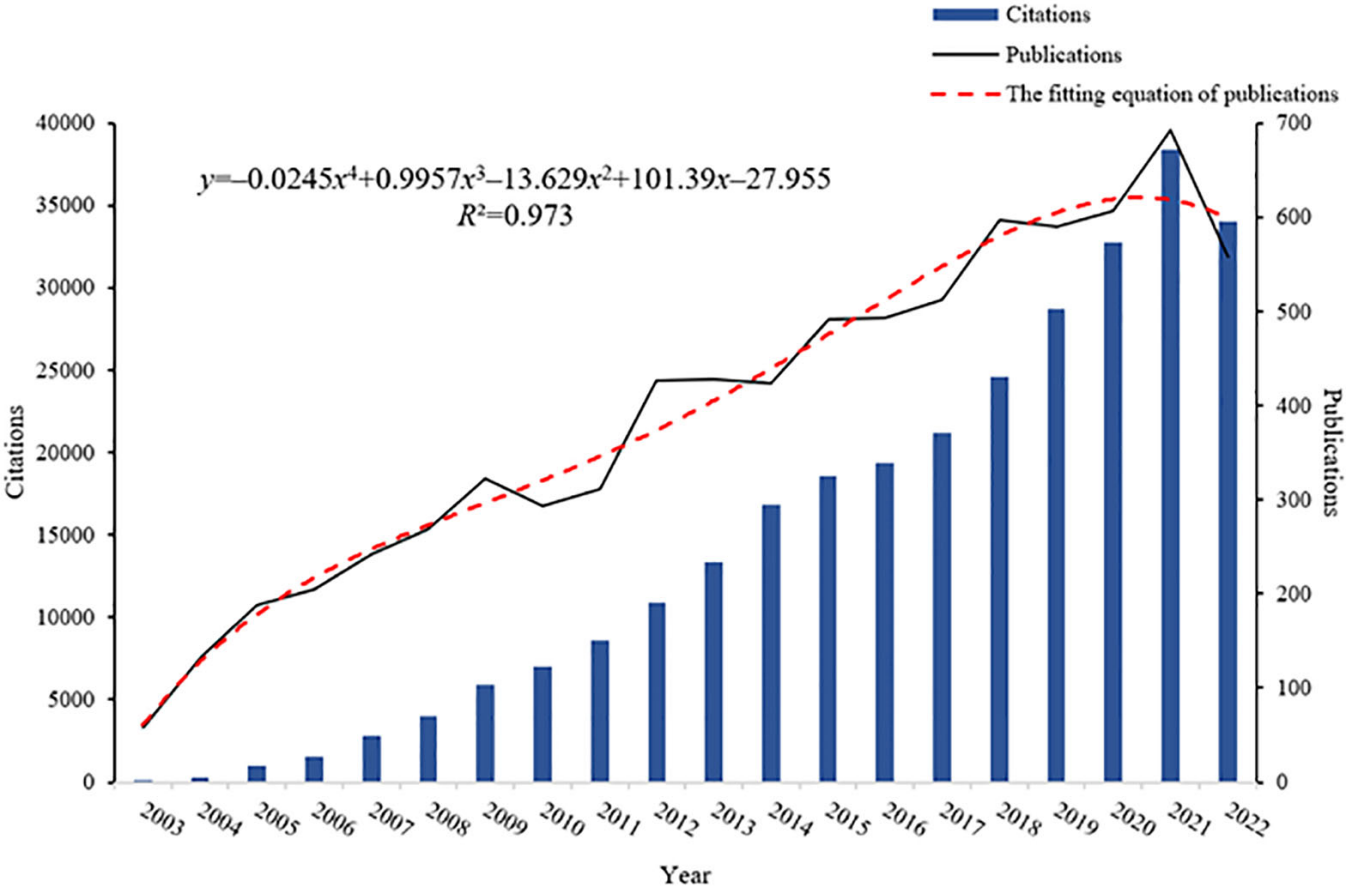
Annual publications, citation counts, and the fitting equation for annual publications in BPD.

Annual publications can provide an overview of the evolution of a research area and its progress (28). We retrieved 7919 articles from the WOS database on BPD between 2003 and 2022, including 6834 research articles and 1085 reviews (**Figure 1**). As of the search date, these articles had received a total of 289,958 citations, equating to an average of 14,498 citations per year. Over the past two decades, the number of research articles published on BPD has shown a fluctuating upward trend. In addition, citations to these publications have increased significantly. A polynomial curve fit of the literature on BPD clearly indicates a strong correlation between the year of publication and the number of publications (*R* ^2 =^ 0.973). The number of research articles on BPD has indeed fluctuated and increased over the past two decades. This observation does, to some extent, indicate an upward trend, probably due to increasing interest in BPD. However, there are other factors to consider as well. For example, the accumulation of data or technological advances, government policies and corporate investment may also affect the direction of BPD research development.

### 3.2 Analysis of co-citation references: clusters and timeline of research

**Figure 2:**
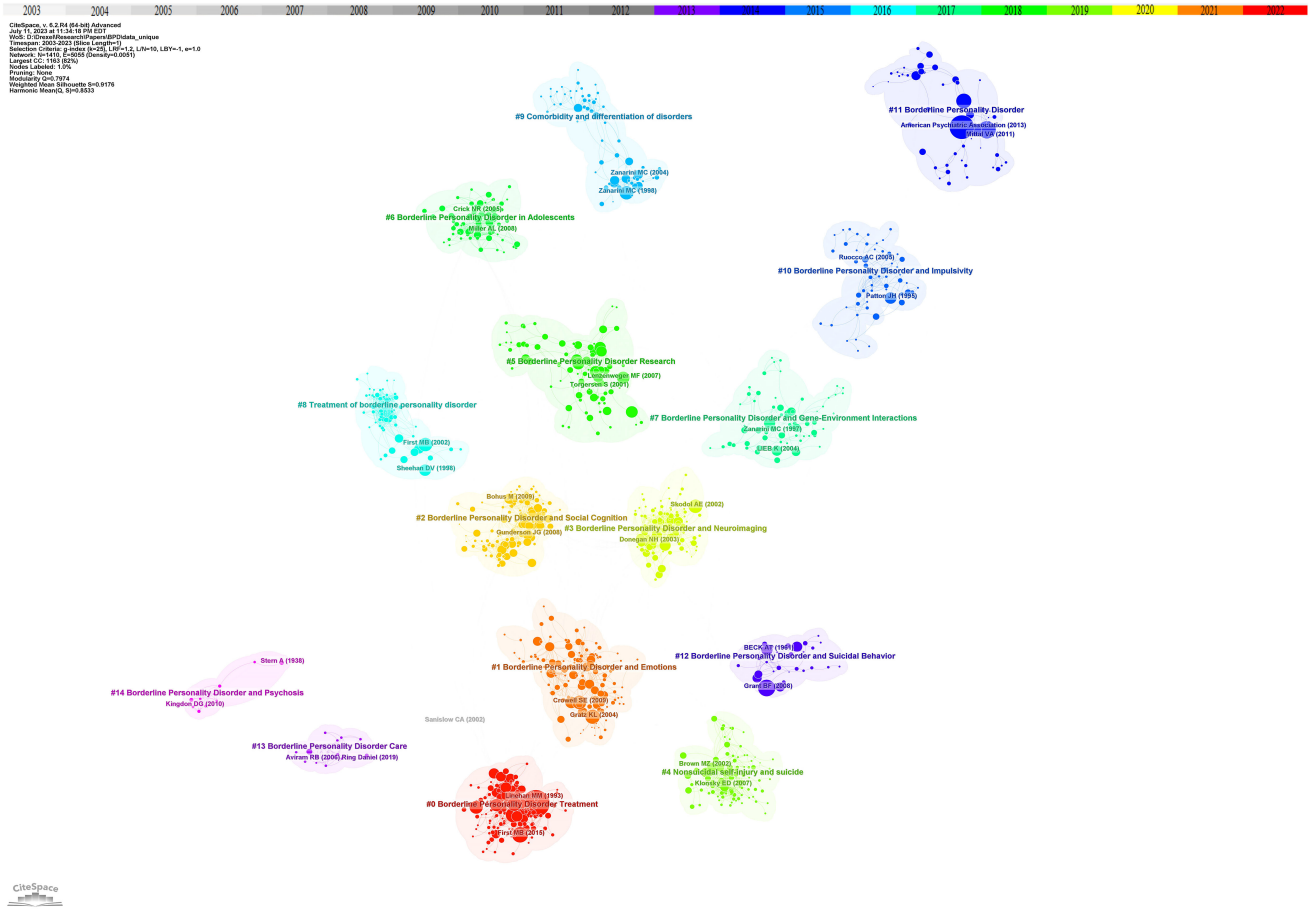
Reference co-citation network with cluster visualization in BPD. Trend 1 clinical researches, sub-trend clinical characteristics includes clusters #1, #2, #4, #10, #12; biological mechanisms include clusters #3, #7; nursing treatments includes clusters #0, #8, #13. Trend 2 associations and complications includes clusters #5, #6, #9, #11, #14.

Co-cited references, which are cited by multiple papers concurrently, are considered a crucial knowledge base in any given field (28). In the current study, CiteSpace clustering was utilized to identify common themes within BPD-related literature. **Figure 2** presented a co-citation network of highly cited references between 2003 and 2022, comprising 1163 references. A time slice of 1 was used, with the *g*-index was set at *k*=25, which resulted in the identification of 14 clusters representing distinct research themes in BPD. The significant cluster structure is denoted by a modularity value (*Q* value) of 0.7974, and the high confidence level in the clusters by an average profile value (*S* value) of 0.9176.

Cluster analysis is performed through CiteSpace. Related clusters are classified into the same trend based on the knowledge of related fields and whether the clusters show similar trends. At the same time, based on the analysis of time series, to identify the movement of one cluster to another. Based on the cluster map of co-cited references on BPD, several different research trends were identified. The first major research trend is clinical research on BPD, which in turn consists of three sub-trends: clinical characterization of BPD, biological mechanisms, and nursing treatment. Of the data obtained, the earliest research on the clinical characterization of BPD began in 1992 with cluster #12, “borderline personality disorder and suicidal behavior” (*S*=0.979; 1992). Paul H. Soloff and his colleagues conducted a comparative study of suicide attempts between major depressives and patients with BPD. The aim of this study was to develop more effective intervention strategies for suicide prevention (29). This cluster was further developed in cluster #4, “nonsuicidal self-injury and suicide” (*S*=0.96; 2004). Thomas A. Widiger and Timothy J. Trull proposed a more flexible dimension-based categorization model to overcome the previous drawbacks of personality disorder categorization (30). Next in cluster #10 “borderline personality disorder and impulsivity” (*S*=0.93; 2000), Jim H. Patton and his colleagues revised the Barratt Impulsivity Scale to measure impulsivity to facilitate practical clinical research (31). Related research continues to evolve into cluster #1 “borderline personality disorder and emotions” (*S*=0.87; 2007) and cluster #2 “borderline personality disorder and social cognition” (*S*=0.911; 2009), researchers have focused on understanding the causal relationship between BPD traits and factors such as social environment, emotion regulation, and interpersonal evaluative bias, as well as their potential impact (32, 33). In the sub-trend of biological mechanisms, two main clusters are involved: cluster #7 “borderline personality disorder and gene-environment interactions” (*S*=0.871; 2002) and cluster #3 “borderline personality disorder and neuroimaging” (*S*=0.938; 2007). In the related cluster, researchers have found a relationship between BPD and genetic and environmental factors (34). Researchers have also utilized various external techniques to explore the degree of correlation between the risk of developing BPD and its biological mechanisms, aiming to reveal the complex mechanisms that influence the emergence and development of BPD (35). In nursing treatment, cluster #8 “treatment of borderline personality disorder “ (*S*=0.968; 2001), Silvio Bellino and his colleagues systematically analyzed the current publications on BPD pharmacotherapy research and summarized relevant clinical trials and findings (36). However, due to the complexity of BPD, there is still a lack of information on the exact efficacy of pharmacotherapy in BPD, and therefore pharmacotherapy remains an area of ongoing development and research. This trend continues to be developed in cluster #0 “borderline personality disorder treatment” (*S*=0.887; 2006), which emphasizes the development of novel pharmacotherapies for BPD. Cluster #13 “borderline personality disorder care” (*S*=0.997; 2013) mainly focuses on the comprehensive care of people with borderline personality disorder and the education of patients and families. The goal is to improve patients’ quality of life, reduce self-injury and suicidal behavior, and promote full recovery.

The second major research trend is association and comorbidity. This trend first began in cluster #9 “comorbidity and differentiation of disorders” (*S*=0.946; 1999). Mary C Zanarini and his colleagues explored the comorbidity of BPD with other psychiatric disorders on Axis I (37). Cluster #14 “borderline personality disorder and psychosis” (*S*=0.966; 2003) also explored symptoms associated with BPD (38). This trend continues, with researchers studying BPD research in cluster #11 “borderline personality disorder” (*S*=0.935; 2004) and cluster #5 “borderline personality disorder research” (*S*=0.881; 2007) (39, 40). In addition, cluster #6 “borderline personality disorder in adolescents” (*S*=0.894; 2011) points out that the focus of BPD research is increasingly shifting towards adolescents (41).

**Figure 3:**
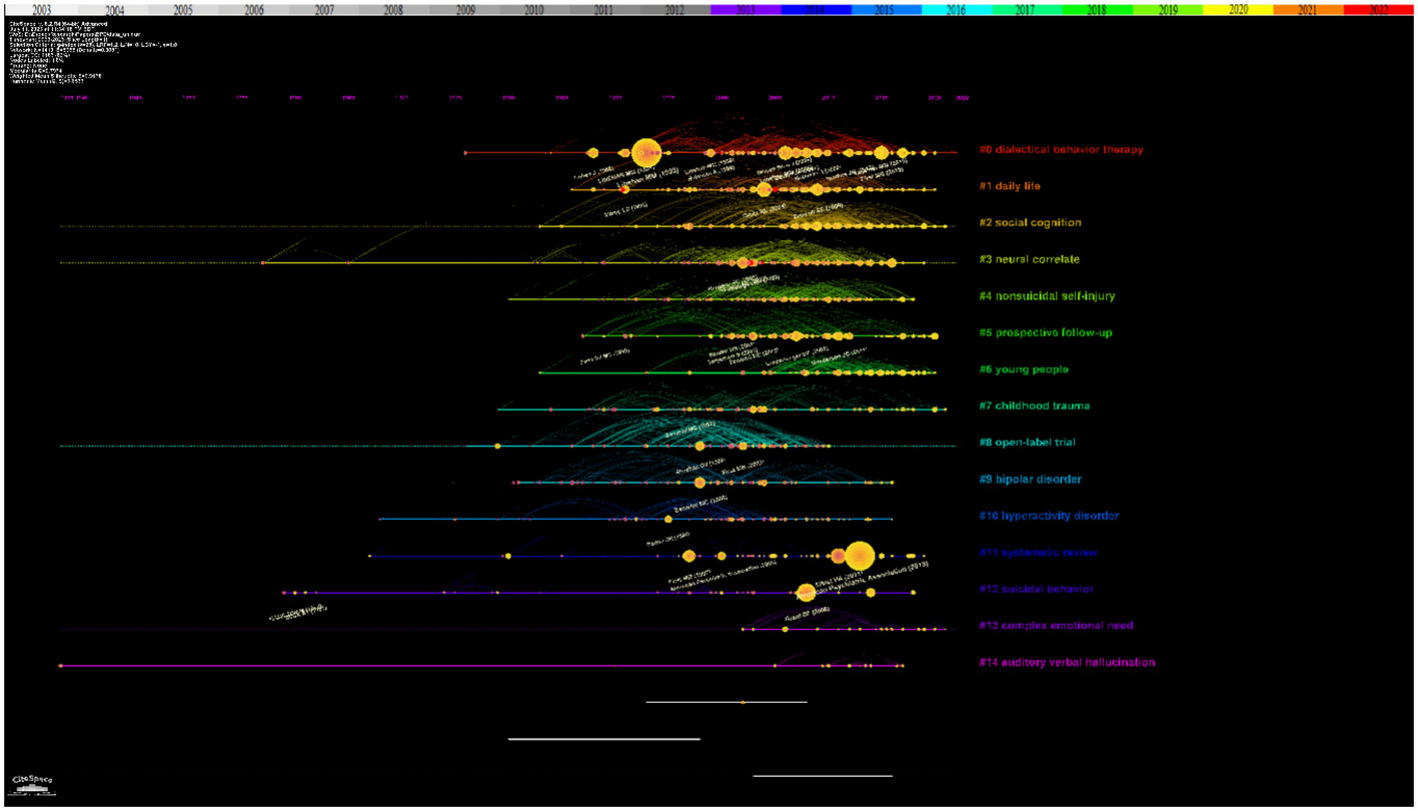
Reference co-citation network with timeline visualization in BPD.

**Figure 3** showed the time span and research process of the developmental evolution of these different research themes. The temporal view reveals the newest and most active clusters, namely #0 “dialectical behavior therapy”, #1 “daily life”, and #2 “social cognition”, which have been consistently researched for almost a decade. Cluster #0 “dialectical behavior therapy” has the largest number and the longest duration, lasting almost 10 years. Similarly, this article by Rebekah Bradley and Drew Westen on understanding the psychodynamic mechanisms of BPD from the perspective of developmental psychopathology has the largest node (34).

### 3.3 Most cited papers

The top 10 highly cited papers on BPD research were presented in **Table 1**. The most cited paper, by Marsha M. Linehan and colleagues, focus on the treatment of suicidal behavior in BPD (42). The transition between suicidal and non-suicidal self-injurious behavior in individuals with BPD has attracted researchers’s attention, mainly in cluster #4 “nonsuicidal self-injury and suicide” (52). The second is the experimental study by Josephine Giesen-Bloo and his colleagues on the psychotherapy of BPD (43). In cluster #0 “borderline personality disorder treatment” and Cluster #8 “treatment of borderline personality disorder”, researchers strive to find non-pharmacological approaches with comparable or enhanced therapeutic effects. This was followed by Sheila E. Crowell and her colleagues’ study of the biological developmental patterns of BPD (44). Research on the biological mechanisms and other contributing factors of BPD, including #7 “borderline personality disorder and gene-environment interactions” have been closely associated with the development of BPD (53).

**Table 1:** Top 10 cited references that published BPD researches.

Rank	Citation (WOS/Google Scholar)	Cited reference and year	Title	Journals
1	1193/2771	Linehan et al. (42),	Two-year randomized controlled trial and follow-up of dialectical behavior therapy vs therapy by experts for suicidal behaviors and borderline personality disorder	Archives of General Psychiatry
2	715/1742	Giesen-Bloo et al. (43),	Outpatient psychotherapy for borderline personality disorder - Randomized trial of schema-focused therapy vs transference-focused psychotherapy	Archives of General Psychiatry
3	711/1475	Crowell et al. (44),	A biosocial developmental model of borderline personality: Elaborating and extending Linehan’s theory	Psychological Bulletin
4	591/1240	Leichsenring et al., (45)	Borderline personality disorder	Lancet
5	585/1068	Whiteside et al., (46)	Validation of the UPPS impulsive behaviour scale: a four-factor model of impulsivity	European Journal of Personality
6	581/1517	Clarkin et al., (47)	Evaluating three treatments for borderline personality disorder: A multiwave study	The American Journal of Psychiatry
7	560/1220	Bateman & Fonagy, (48)	Randomized controlled trial of outpatient mentalization-based treatment versus structured clinical management for borderline personality disorder randomized controlled trial of outpatient mentalization-based treatment versus structured clinical management for borderline personality disorder	The American Journal of Psychiatry
8	550/1278	Fonagy et al., (49)	A developmental, mentalization-based approach to the understanding and treatment of borderline personality disorder	Development and Psychopathology
9	517/1207	Levy et al., (50)	Change in attachment patterns and reflective function in a randomized control trial of transference-focused psychotherapy for borderline personality disorder	Journal of Consulting and Clinical Psychology
10	492/902	Carr et al., (51)	8-year follow-up of patients treated for borderline personality disorder: Mentalization-based treatment versus treatment as usual	The American Journal of Psychiatry

### 3.4 Burst analysis and transformative papers

**Figure 4:**
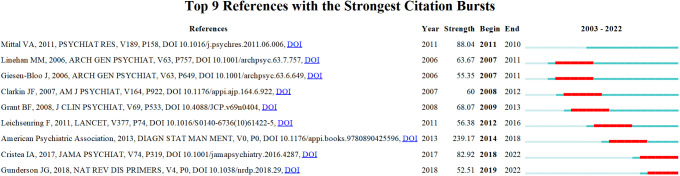
References with the strongest occurrence burst on BPD researches. Article titles correspond from top to bottom: Mittal VA et al. Diagnostic and Statistical Manuel of Mental Disorders; Linehan MM et al. Two-year randomized controlled trial and follow-up of dialectical behavior therapy vs therapy by experts for suicidal behaviors and borderline personality disorder; Giesen-Bloo J et al. Outpatient psychotherapy for borderline personality disorder: Randomized trial of schema-focused therapy vs transference-focused psychotherapy; Clarkin Jf et al. Evaluating three treatments for borderline personality disorder: A multiwave study; Grant BF et al. Prevalence, correlates, disability, and comorbidity of DSM-IV borderline personality disorder: Results from the Wave 2 National Epidemiologic Survey on Alcohol and Related Conditions; Leichsenring F et al. Borderline personality disorder; American Psychiatric Association, DSM-5 Task Force. Diagnostic and statistical manual of mental disorders: DSM-5™ (5th ed.); Cristea IA et al. Efficacy of psychotherapies for borderline personality disorder: A systematic review and meta-analysis; Gunderson JG et al. Borderline personality disorder.

The “citation explosion” reflects the changing research focus of a field over time and indicates that certain literature has been frequently cited over time. **Figure 4** showed the top 9 references with the highest citation intensity. The three papers with the greatest intensity of outbursts during the period 2003−2022 are: The first is the American Psychiatric Association’s Diagnostic and Statistical Manual of Mental Disorders (54). In the second article, Vijay A. Mittal and Elaine F. Walker discuss key issues surrounding dyspraxia, tics, and psychosis that are likely to appear in an upcoming edition of the Diagnostic and Statistical Manual of Mental Disorders (39). In addition, Ioana A. Cristea and colleagues conducted a systematic review and meta-analysis to evaluate the effectiveness of psychotherapy for borderline personality disorder (55).

Structural variation analysis can be understood as a method of measuring and studying structural changes in the field, mainly reflecting the betweenness centrality and sigma of the references. The high centrality of the reference plays an important role in the connection between the preceding and following references and may help to identify critical points of transformation, or intellectual turning points. Sigma values, on the other hand, are used to measure the novelty of a study, combining a combination of citation burst and structural centrality (56). **Table 2** listed the top 10 structural change references that can be considered as landmark studies connecting different clusters. The top three articles with high centrality are the studies conducted by Milton Z. Brown and his colleagues on the reasons for suicide attempts and non-suicidal self-injury in BPD women (57); the research by Nelson H. Donegan and his colleagues on the impact of amygdala on emotional dysregulation in BPD patients (59); and the fMRI study by Sabine C. Herpertz and her colleagues on abnormal amygdala function in BPD patients (61). In addition, publications with high sigma values are listed. They are Larry J. Siever and Kenneth L. Davis on psychobiological perspectives on personality disorders (58); Ludger Tebartz van Elst and his colleagues on abnormalities in frontolimbic brain functioning (60); and Marsha M. Linehan on therapeutic approaches in BPD research (62). These works are recognized as having transformative potential and may generate some new ideas.

**Table 2:** Top 7 betweenness centrality and stigma references.

References	Betweenness Centrality	References	Sigma
Brown et al. (57), J ABNORM PSYCHOL	0.07	Siever and Davies (58), AM J PSYCHIAT	2.71
Donegan et al. (59), BIOL PSYCHIAT	0.06	van Elst et al. (60), BIOL PSYCHIAT	1.30
Herpertz et al. (61), BIOL PSYCHIAT	0.05	Linehan MM (62),, ARCH GEN PSYCHIAT	1.28
Black DW, (63), J PERS DISORD	0.05	Brambilla P, (64), PSYCHIAT RES-NEUROIM	1.16
Crick et al. (41), DEV PSYCHOPATHOL	0.04	Stiglmayr CE, (65), ACTA PSYCHIAT SCAND	1.09
Silbersweig D, (66), AM J PSYCHIAT	0.04	Bohus M, (67), BEHAV RES THER	1.04
Rossouw TI, (68), J AM ACAD CHILD PSY	0.04	Brown et al. (57), J ABNORM PSYCHOL	1.00

### 3.5 Analysis of authors and co-authors

**Figure 5:**
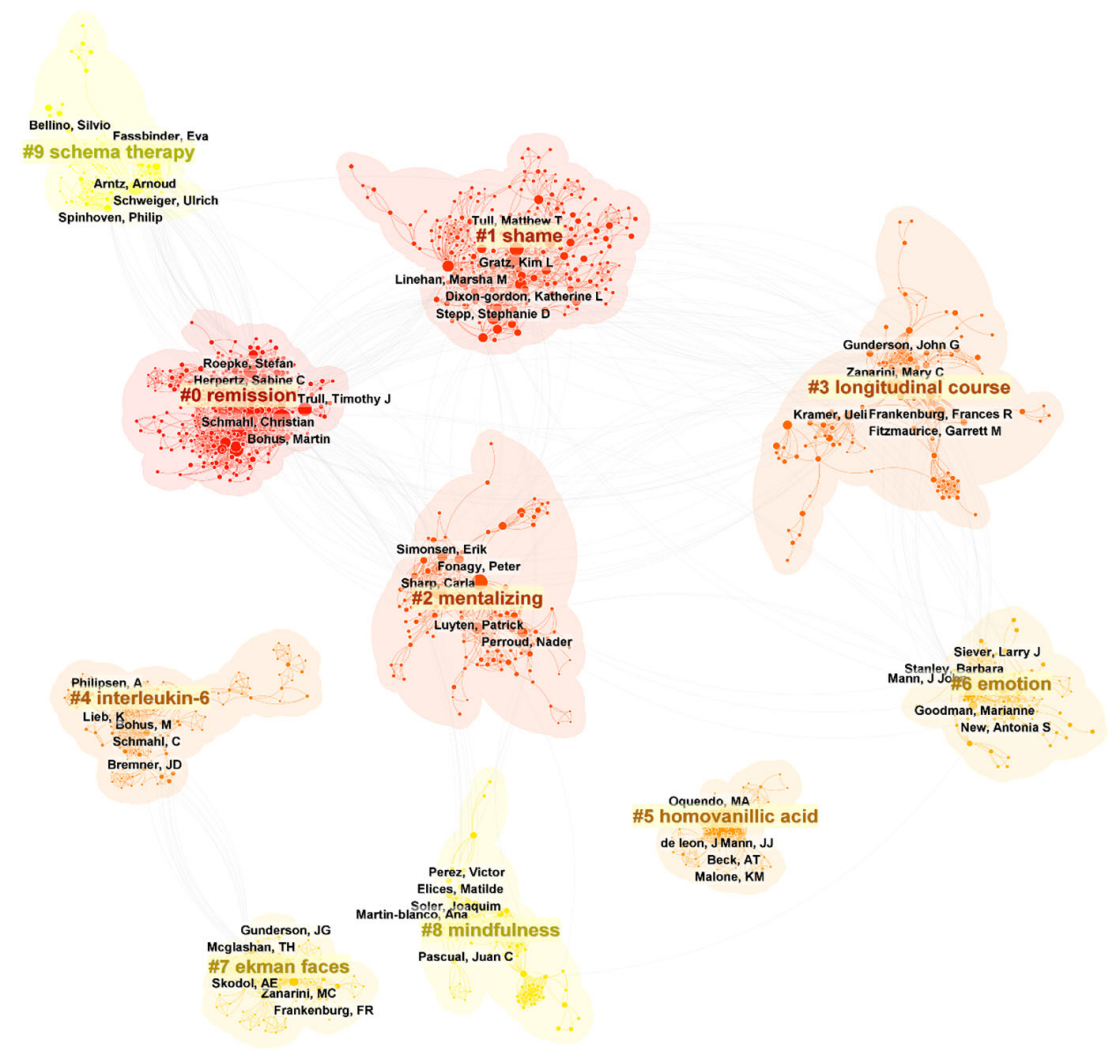
Top 10 clusters of coauthors in BPD (2003–2023). Selection Criteria: Top 10 per slice. Clusters labeled by keywords. The five authors with the highest number of publications in each cluster were labeled.

**Figure 5** showed a map of the co-authorship network over the last two decades. In total, 10 different clusters are shown, each of which gathers co-authors around the same research topic. For example, the main co-authors of cluster #0 “remission” are Christian Schmahl, Martin Bohus, Sabine C. Herpertz, Timothy J. Trull and Stefan Roepke. More recently, the three authors with the greatest bursts of research have been Mary C. Zanarini, Erik Simonsen, and Carla Sharp. As shown in **Table 3**, the three most published authors are Martin Bohus (145 publications; 1.83%; H-index=61), Mary C. Zanarini (144 publications; 1.82%; H-index=80) and Christian Schmahl (142 publications; 1.79%; H-index=54).

**Table 3:** Top 10 authors that published BPD researches.

Rank	Authors	Publications (%)	Institution	Country	H–index
1	Martin Bohus	145 (1.83)	Central Institute of Mental Health	Germany	61
2	Mary C. Zanarini	144 (1.82)	McLean Hospital	The United States	80
3	Christian Schmahl	142 (1.79)	Central Institute of Mental Health	Germany	54
4	Carla Sharp	101 (1.28)	University of the Free State	South Africa	44
5	Peter Fonagy	98 (1.24)	University College London	The United Kingdom	89
6	Kim L. Gratz	92 (1.16)	University of Toledo	The United States	52
7	Arnoud Arntz	88 (1.11)	University of Amsterdam	Netherlands	67
8	Sabine C. Herpertz	87 (1.10)	University of the Free State	South Africa	43
9	Timothy J. Trull	77 (0.97)	University of Missouri Columbia	The United States	15
10	Klaus Lieb	71 (0.90)	Leibniz Institute for Resilience Research	Germany	64

### 3.6 Analysis of cooperation networks across countries

**Figure 6:**
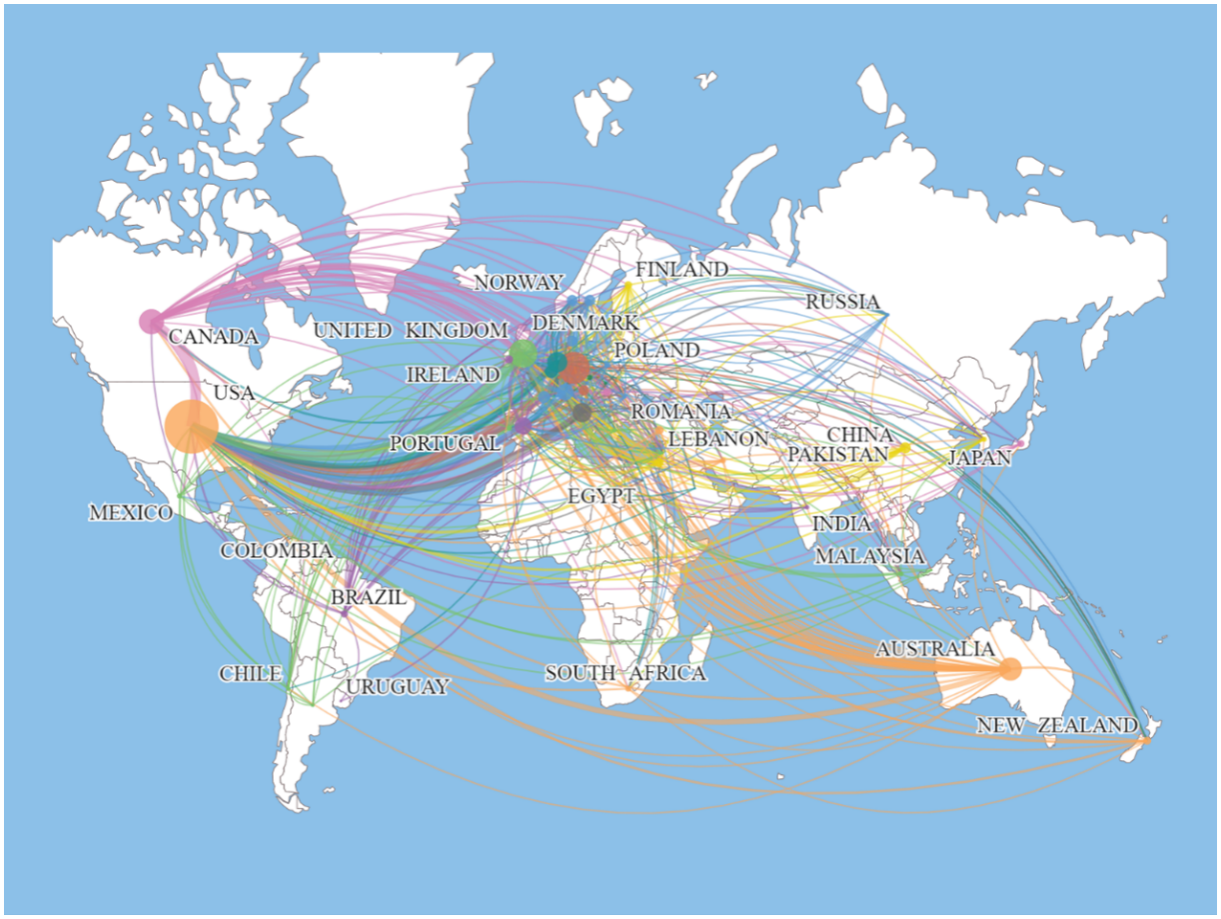
Map of the distribution of countries/regions engaged in BPD researches.

The top 10 countries in terms of number of publications in the BPD are added in **Table 4**. With 3,440 published papers, or nearly 43% of all BPD research papers, the United States is the leading contributor to BPD research. This is followed by Germany (1196 publications; 15.10%) and the United Kingdom (1020 publications; 9.32%). Centrality refers to the degree of importance or centrality of a node in a network and is a measure of the importance of a node in a network (69). In **Table 4** the United States is also has the highest centrality (0.43). **Figure 6** shows the geographic collaboration network of countries in this field, with 83 countries contributing to BPD research, primarily from the United States and Europe.

**Table 4:** Top 10 countries that published BPD researches.

Rank	Country	Publications (%)	Centrality
1	The United States	3444 (43.49)	0.43
2	Germany	1196 (15.10)	0.11
3	The United Kingdom	1021 (12.89)	0.03
4	Canada	738 (9.32)	0.09
5	Australia	627 (7.92)	0.22
6	Netherlands	478 (6.04)	0.03
7	Italy	438 (5.53)	0.06
8	Spain	355 (4.48)	0.14
9	Switzerland	328 (4.14)	0.10
10	Belgium	192 (2.42)	0.02

### 3.7 Analysis of the co-author’s institutions network

**Table 5** listed the top 10 institutions ranked by the number of publications. The current study shows that Research Libraries Uk is the institution with the highest number of publications, with 766 publications (9.67%). The subsequent institutions are Harvard University and Ruprecht Karls University Heidelberg with 425 (5.37%) and 389 (4.91%) publications respectively. As can be seen from **Table 4**, six of the top 10 institutions in terms of number of publications are from the United States. In part, this reflects the fact that the United States institutions are at the forefront of the BPD field and play a key role in it.

**Table 5:** Top 10 institutions that published BPD researches.

Rank	Institution	Publications (%)	Country
1	Research Libraries Uk	766 (9.67)	The United Kingdom
2	Harvard University	425 (5.37)	The United States
3	Ruprecht Karls University Heidelberg	389 (4.91)	Germany
4	University of London	382 (4.82)	The United Kingdom
5	Pennsylvania Commonwealth System of Higher Education Pcshe	314 (3.97)	The United States
6	Central Institute of Mental Health	291 (3.67)	Germany
7	Mclean Hospital	271 (3.42)	The United States
8	Department of Veterans Affairs	244 (3.08)	The United States
9	Veterans Health Administration	242 (3.06)	The United States
10	Columbia University	229 (2.89)	The United States

### 3.8 Analysis of journals and cited journals

If the more papers are published in a particular journal and at the same time it has a high number of citations, then it can be considered that the journal is influential (70). The top 10 journals in the field of BPD in terms of number of publications are listed in **Table 6**. *Journal of Personality Disorders* from the Netherlands published the most literature on BPD with 438 (5.53%; IF=3.367) publications. This was followed by two journals from the United States: *Psychiatry Research* and *Personality Disorders Theory Research and Treatment*, with 269 (3.40%, IF=11.225) and 232 (2.93%; IF=4.627) publications, respectively. Among the top 10 journals in terms of number of publications published, *Psychiatry Research* has the highest impact factor.

**Table 6:** Top 10 journals that published BPD researches.

Journals	Publications (%)	Country	IF (2021)
Journal of Personality Disorders	438 (5.53)	Netherlands	3.367
Psychiatry Research	269 (3.40)	The United States	11.225
Personality Disorders Theory Research and Treatment	232 (2.93)	The United States	4.627
Journal of Affective Disorders	200 (2.53)	Netherlands	6.533
Frontiers in Psychiatry	124 (1.58)	Switzerland	5.435
Comprehensive Psychiatry	123 (1.55)	The United States	7.211
Personality and Mental Health	121 (1.53)	The United Kingdom	3.304
Borderline Personality Disorder and Emotion Dysregulation	118 (1.49)	The United Kingdom	4.463
Journal of Clinical Psychology	115 (1.45)	The United States	2.995
Psychological Medicine	109 (1.38)	The United Kingdom	10.592

### 3.9 Analysis of keywords and keywords co-occurrence

**Figure 7:**
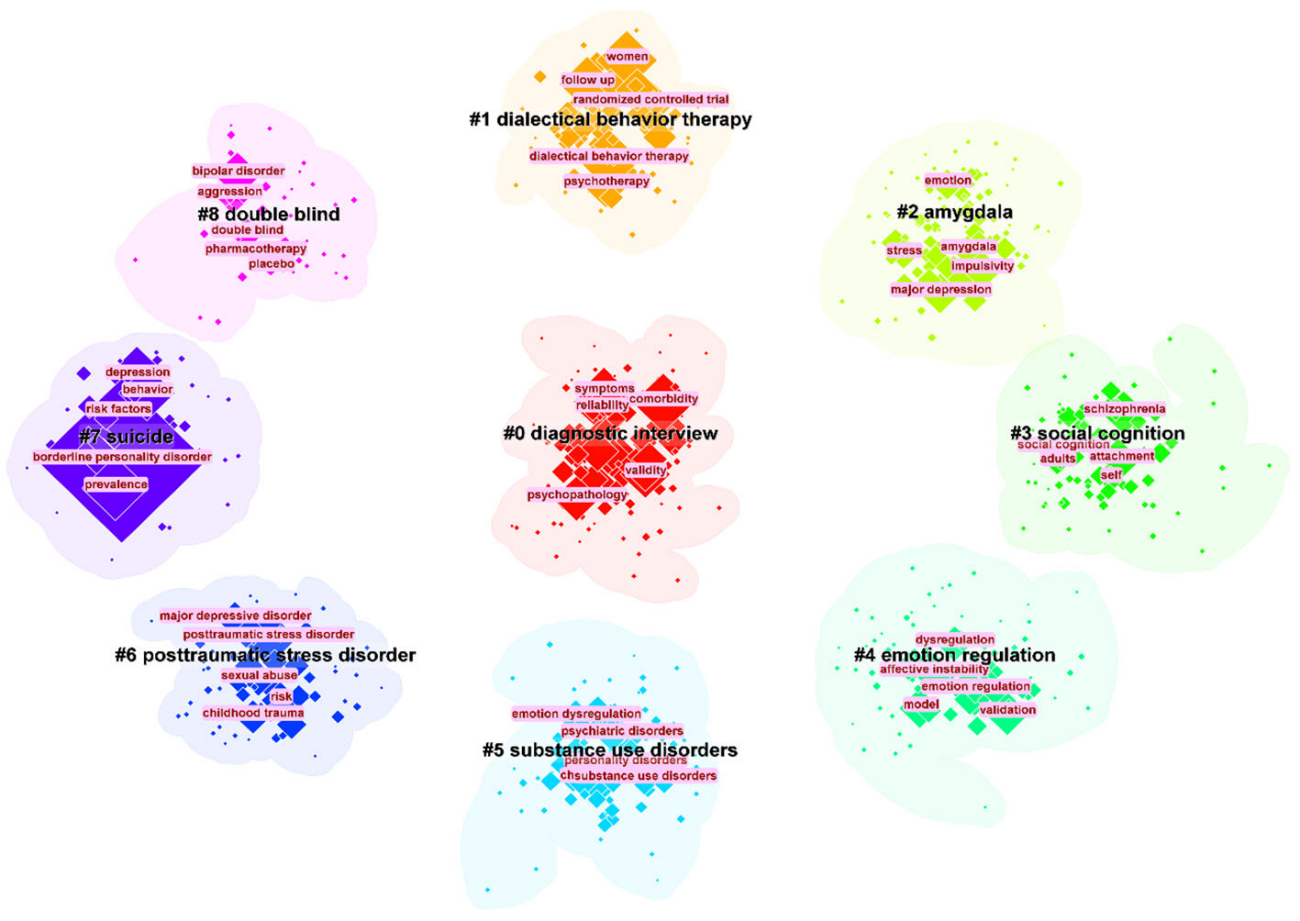
The largest 9 clusters of co-occurring keywords. The top 5 most frequent keywords in each cluster are highlighted.

Keyword co-occurrence analysis can help researchers to understand the research hotspots in a certain field and the connection between different research topics. As shown in **Figure 7**, all keywords can be categorized into 9 clusters: cluster #0 “diagnostic interview”, cluster #1 “diagnostic behavior therapy”, cluster #3 “social cognition”, cluster #4 “emotional regulation”, cluster #5 “substance use disorders “, cluster #6 “posttraumatic stress disorder”, cluster #7 “suicide” and cluster #8 “double blind”. These keywords have all been important themes in BPD research during the last 20 years.

**Figure 8:**
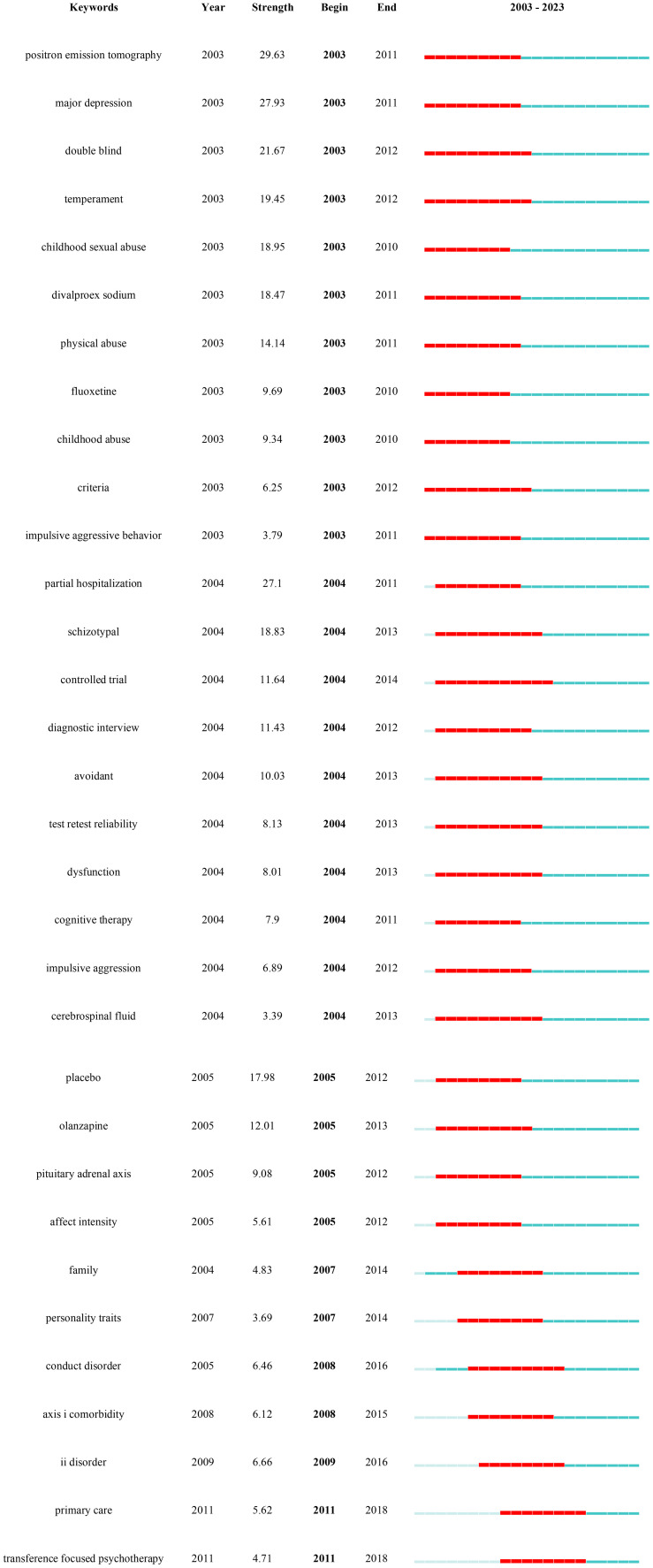
Keywords with the strongest occurrence burst on BPD researches.

Keyword burst is used to identify keywords with a significant increase in the frequency of occurrence in a topic or domain, helping to identify emerging concepts, research hotspots or keyword evolutions in a specific domain (71). **Figure 8** presented the top 32 keywords with the strongest citation bursts in BPD from 2003−2023. Significantly, the keywords “positron emission tomography” (29.63), “major depression” (27.93), and “partial hospitalization” (27.1) had the highest intensity of outbreaks.

## 4 Discussion

### 4.1 Application of the “neuro-behavioral model” to BPD research

In this study, we chose specific search terms, particularly “Neuro-behavioral Model”, to efficiently collect and analyze BPD research literature related to this emerging framework. This choice of keyword helped narrow the research scope and ensure its relevance to our objectives. However, it may have excluded some studies using different terminology, thus limiting comprehensiveness. In addition, the ‘Neuro-behavioral Model’, as an interdisciplinary field, encompasses a wide range of connotations and extensions, which also poses challenges to our research. This undoubtedly adds to the complexity of the study, yet it enhances our understanding of the field’s diversity.

### 4.2 Summary of the main findings

This current study utilized CiteSpace and Scimago Graphic software to conduct a comprehensive bibliometric analysis of the research literature on BPD. The study presented the current status of research, research hotspots, and research frontiers in BPD over the past 20 years (2003–2022) through knowledge mapping. The scientific predictions of future trends in BPD provided by this study can guide researchers interested in this field. This study also uses bibliometrics analysis method to show the knowledge structure and research results in the field of BPD, as well as the scientific prediction of the future trend of BPD research.

### 4.3 Identification of research hotspots

Previous studies have indicated an increasing trend in the number of papers focused on BPD, with the field gradually expanding into various areas. The first major research trend involves clinical studies on BPD. This includes focusing on emotional recognition difficulties in BPD patients, as well as studying features related to suicide attempts and non-suicidal self-injury. Clinical recognition and confirmation of BPD remains low, mainly related to the lack of clarity of its biological mechanisms (72). The nursing environment for BPD patients plays an important role in the development of the condition, which has become a focus of research. Researchers are also exploring the expansion of treatment options from conventional medication to non-pharmacological approaches, particularly cognitive-behavioral therapy. Another major research trend involves the associations and complications of BPD, including a greater focus on the adolescent population to reduce the occurrence of BPD starting from adolescence. Additionally, many researchers are interested in the comorbidity of BPD with various clinical mental disorders.

### 4.4 Potential trends of future research on BPD

Based on the results of the above studies and the results of the research trends in the table of details of the co-citation network clusters in 2022 (**Table 7**), several predictions are made for the future trends in the field of BPD. In **Table 7**, there were some trends related to previous studies, including #1”dialectical behavior therapy”, #7 “dialectical behavior therapy” (73), #5 “mentalization” (74), and #9 “non-suicidal self-injury” (75). The persistence of these research trends is evidence that they have been a complex issue in this field and a focus of researchers. The recently emerged turning point paper provides a comprehensive assessment about BPD, offering practical information and treatment recommendations (76). New research is needed to improve standards and suggest more targeted and cost-effective treatments.

**Table 7:** The references co-citation network cluster detail (2022).

Cluster ID	Size	Silhouette Score	Mean year	Top terms based on keywords (Log-likelihood ratio algorithm; p-level)
0	53	0.862	2010	**oxytocin (11.86, 0.001)**; childhood maltreatment (11.12, 0.001); dialectical behavior therapy (10.23, 0.005); childhood trauma (9.38, 0.005); emotion recognition (8.78, 0.005)
1	43	0.913	2009	**dialectical behavior therapy (14.12, 0.001)**; psychotherapy (9.13, 0.005); personality disorder (6.6, 0.05); follow-up (5.22, 0.05); depression (4.93, 0.05)
2	34	0.897	2008	**bipolar disorder (7, 0.01)**; psychiatry (6.58, 0.05); major depression (4.96, 0.05); diagnosis (4.96, 0.05); neuroticism (4.96, 0.05)
3	28	0.909	2012	**youth (10.77, 0.005)**; adolescents (9.36, 0.005); adolescence (6.61, 0.05); developmental psychopathology (6.4, 0.05); young people (6.4, 0.05)
4	26	0.875	2012	**stigma (7.77, 0.01)**; health services (6.74, 0.01); structural stigma (6.74, 0.01); crisis care (6.74, 0.01); mental health services (5.2, 0.05)
5	25	0.95	2008	**mentalization (26.66, 1.0E-4)**; reflective functioning (21.69, 1.0E-4); mentalizing (16.13, 1.0E-4); theory of mind (10.74, 0.005); borderline personality disorder (7.65, 0.01)
6	20	0.827	2006	**icd-11 (15.55, 1.0E-4)**; classification (10.36, 0.005); alternative model for personality disorders (ampd) (10.36, 0.005); severity (10.36, 0.005); disinhibition (10.36, 0.005)
7	16	0.884	2008	**dialectical behaviour therapy (11.99, 0.001)**; bulimia nervosa (10.66, 0.005); emotion regulation (10.35, 0.005); emotion dysregulation (9.47, 0.005); telehealth (6.53, 0.05)
8	16	0.861	2007	**relatives (11.18, 0.001)**; alcohol dependence (6.45, 0.05); family connections (6.45, 0.05); alcohol (6.45, 0.05); caregivers (6.45, 0.05)
9	15	0.979	2009	**non-suicidal self-injury (22.52, 1.0E-4)**; non-suicidal self-injury (22.21, 1.0E-4); ecological momentary assessment (10.48, 0.005); self-injury (10.16, 0.005); self-harm (8.7, 0.005)
10	4	0.995	2007	**parenting (20.14, 1.0E-4)**; coding interactive behavior (8.14, 0.005); authoritarian parenting style (8.14, 0.005); prevalence (8.14, 0.005); thematic analysis (8.14, 0.005)

BPD symptoms in adolescents have been shown to respond to interventions with good results, so prevention and intervention for BPD is warranted (77). This trend can be observed in #3 “youth” (78). Mark F. Lenzenweger and Dante Cicchetti summarized the developmental psychopathology approach to BPD, one of the aims of which is to provide information for the prevention of BPD (79). Prevention and early intervention of BPD has been shown to provide many benefits, including reduced occurrence of secondary disorders, improved psychosocial functioning, and reduced risk of interpersonal conflict (80). However, there are differences between individuals, and different prevention goals are recommended for adolescents at risk for BPD. Therefore, prevention and early intervention for BPD has good prospects for the future.

The etiology of BPD is closely related to many factors, and its pathogenesis is often ignored by clinicians. The exploration of risk factors has been an important research direction in the study. Some studies have found that BPD is largely the product of traumatic childhood experiences, which may lead to negative psychological effects on children growing up (81). It has also been found that the severity of borderline symptoms in parents is positively associated with poor parenting practices (82). Future researches need to know more about the biological-behavioral processes of parents in order to provide targeted parenting support and create a good childhood environment.

Because pharmacotherapy is only indicated for comorbid conditions that require medication, psychotherapy has become one of the main approaches to treating BPD. The increasingly advanced performance and availability of contemporary mobile devices can help to take advantage of them more effectively in the context of optimizing the treatment of psychiatric disorders. The explosion of COVID-19 is forcing people to adapt to online rather than face-to-face offline treatment (83). The development of this new technology will effectively advance the treatment of patients with BPD. Although telemedicine has gained some level of acceptance by the general public, there are some challenges that have been reported, so further research on the broader utility of telemedicine is needed in the future.

### 4.5 The current study compares with a previous bibliometric review of BPD

As mentioned earlier, there have been previous bibliometric studies conducted by scholars in the field of BPD. This paper focuses more on BPD in personality disorders than the extensive study of personality disorders as a category by Taylor Reis et al. (15). The results of both studies show an increasing trend in the number of publications in the field of BPD, suggesting positive developments in the field. Taylor Reis et al. focused primarily on quantifying publications on personality disorders and did not delve into other specific aspects of BPD. Ilaria M.A. Benzi et al. focused on a bibliometric analysis of the pathology of BPD (14). They give three trends for the future development of BPD pathology: first, the growing importance of self-injurious behavior research; second, the association of attention deficit hyperactivity disorder with BPD and the influence of genetics and heritability on BPD; and third, the new focus on the overlap between fragile narcissism and BPD. The study in this paper also concludes that there are three future development directions for BPD: first, the prevention and early intervention of BPD; second, the non-pharmacological treatment of BPD; and third, research into the pathogenesis of BPD. Owing to variations in research backgrounds and data sources, the outcomes presented in the two studies diverge significantly. Nevertheless, both contributions hold merit in advancing the understanding of BPD. In addition to this, this paper also identifies trends in BPD over the past 20 years: the first trend is the clinical research of BPD, which is specifically subdivided into three sub-trends; the second trend is association and comorbidity. The identification of these trends is important for understanding the disorder, improving diagnosis and treatment, etc. Structural variant analysis also features prominently in the study. The impact of literature in terms of innovativeness is detected through in-depth mining and analysis of large amounts of literature data. This analysis is based on research in the area of scientific creativity, especially the role and impact of novel reorganizations in creative thinking. Structural variation analysis is precisely designed to find and reveal embodiments of such innovative thinking in scientific literature, enabling researchers to more intuitively grasp the dynamics and cutting-edge advances in the field of science.

## 5 Limitations

However, it must be admitted that our study has some limitations. The first is the limited nature of data resources. The data source for our study came from only one database, WOS. Second, the limitation of article type. Search criteria are limited to papers and reviews in SCI and SSCI databases. Third, the effect of language type. In the current study, only English-language literature could be included in the analysis, which may lead us to miss some important studies published in other languages. Fourth, limitations of research software. Although this study used well-established and specialized software, the results obtained by choosing different calculation methods may vary. Finally, the diversity of results interpretation. The results analyzed by the software are objective, but there is also some subjectivity in the interpretation and analysis of the research results. While we endeavor to be comprehensive and accurate in our research, the choice of search terms inevitably introduces certain limitations. Using “Neuro-behavioral Model” as the search term enhances the study’s relevance, but it may also cause us to miss significant studies in related areas. This limits the generalizability and replicability of our results. Furthermore, the inherent complexity and diversity of neurobehavioral models might introduce subjectivity and bias in our interpretation and application of the literature. Although we endeavored to reduce bias via multi-channel validation and cross-referencing, we cannot entirely eliminate its potential impact on our findings.

## 6 Conclusion

Overall, a comprehensive scientometrics analysis of BPD provides a comprehensive picture of the development of this field over the past 20 years. This in-depth examination not only reveals research trends, but also allows us to understand which areas are currently hot and points the way for future research efforts. In addition, this method provides us with a framework to evaluate the value of our own research results, which helps us to more precisely adjust the direction and strategy of research. More importantly, this in-depth analysis reveals the depth and breadth of BPD research, which undoubtedly provides valuable references for researchers to have a deeper understanding of BPD, and also provides a reference for us to set future research goals. In short, this scientometrics approach gives us a window into the full scope of BPD research and provides valuable guidance for future research.

## Author contributions

YL: Data curation, Formal analysis, Investigation, Methodology, Software, Visualization, Writing – original draft, Writing – review & editing. CC: Conceptualization, Data curation, Formal analysis, Investigation, Methodology, Project administration, Resources, Software, Supervision, Validation, Visualization, Writing – original draft, Writing – review & editing. YZ: Validation, Visualization, Writing – review & editing. NZ: Conceptualization, Data curation, Formal analysis, Investigation, Methodology, Project administration, Software, Supervision, Validation, Visualization, Writing – original draft, Writing – review & editing. SL: Conceptualization, Data curation, Formal analysis, Funding acquisition, Investigation, Methodology, Project administration, Resources, Software, Supervision, Validation, Visualization, Writing – original draft, Writing – review & editing.
